# Large dynamic range relative $B_{1}^{+}$ mapping

**DOI:** 10.1002/mrm.25884

**Published:** 2015-08-26

**Authors:** Francesco Padormo, Aaron T. Hess, Paul Aljabar, Shaihan J. Malik, Peter Jezzard, Matthew D. Robson, Joseph V. Hajnal, Peter J. Koopmans

**Affiliations:** ^1^ King's College London, Division of Imaging Sciences and Biomedical Engineering, The Rayne Institute, St Thomas' HospitalLondonUnited Kingdom; ^2^ University of Oxford Centre for Clinical Magnetic Resonance Research, Division of Cardiovascular Medicine, Radcliffe Department of MedicineOxfordUnited Kingdom; ^3^ Centre for Functional Magnetic Resonance Imaging of the Brain, Nuffield Department of Clinical Neurosciences, University of OxfordOxfordUnited Kingdom; ^4^ King's College London, Centre for the Developing Brain, Division of Imaging Sciences and Biomedical Engineering, The Rayne Institute, St Thomas' HospitalLondonUnited Kingdom

**Keywords:** parallel transmission, B1 mapping, ultrahigh field MRI

## Abstract

**Purpose:**

Parallel transmission (PTx) requires knowledge of the $B_{1}^{+}$ produced by each element. However, $B_{1}^{+}$ mapping can be challenging when transmit fields exhibit large dynamic range. This study presents a method to produce high quality relative $B_{1}^{+}$ maps when this is the case.

**Theory and Methods:**

The proposed technique involves the acquisition of spoiled gradient echo (SPGR) images at multiple radiofrequency drive levels for each transmitter. The images are combined using knowledge of the SPGR signal equation using maximum likelihood estimation, yielding an image for each channel whose signal is proportional to the $B_{1}^{+}$ field strength. Relative $B_{1}^{+}$ maps are then obtained by taking image ratios. The method was tested using numerical simulations, phantom imaging, and through in vivo experiments.

**Results:**

The numerical simulations demonstrated that the proposed method can reconstruct relative transmit sensitivities over a wide range of $B_{1}^{+}$ amplitudes and at several SNR levels. The method was validated at 3 Tesla (T) by comparing it with an alternative $B_{1}^{+}$ mapping method, and demonstrated in vivo at 7T.

**Conclusion:**

Relative $B_{1}^{+}$ mapping in the presence of large dynamic range has been demonstrated through numerical simulations, phantom imaging at 3T and experimentally at 7T. The method will enable PTx to be applied in challenging imaging scenarios at ultrahigh field. **Magn Reson Med 76:490–499, 2016. © 2015 The Authors. Magnetic Resonance in Medicine published by Wiley Periodicals, Inc. on behalf of International Society for Magnetic Resonance in Medicine. This is an open access article under the terms of the Creative Commons Attribution License, which permits use, distribution and reproduction in any medium, provided the original work is properly cited.**

## INTRODUCTION

Recent years have seen a drive to produce MRI systems with ever‐higher static magnetic field strength. This has been motivated by increased signal‐to‐noise ratio (SNR) and improved contrast. However, there are significant obstacles that still need to be overcome. One of the most prominent is radiofrequency (RF) transmit field ( $B_{1}^{+}$) inhomogeneity; present because the higher Larmor frequency results in a shorter RF wavelength, which in turn leads to increased constructive and destructive interferences 1.

Many methods have been developed to mitigate $B_{1}^{+}$ inhomogeneity, such as the use of $B_{1}^{+}$ insensitive pulses 2, 3 and $B_{1}^{+}$ insensitive pulse sequences 4. A further alternative is the use of multiple transmit channels, known as parallel transmission (PTx) 5, 6, 7. Many methods have been proposed for $B_{1}^{+}$ inhomogeneity correction using PTx. The most basic is $B_{1}^{+}$ shimming, in which the complex gain (often referred to as RF shims) of the RF pulse transmitted by each channel is optimized to yield the most uniform net $B_{1}^{+}$ field 8, 9. More sophisticated methods involve designing the RF pulse waveforms on a channel‐by‐channel basis to produce an excitation with a more uniform flip angle 10, 11.

All methods which use PTx are predicated upon knowledge of the transmit fields produced by each channel, usually obtained in a calibration step at the start of the experimental session. A multitude of $B_{1}^{+}$ mapping methods have been proposed to measure the amplitude of transmit field (known as $B_{1}^{+}$ mapping; see 12, 13, 14, 15, 16, 17 for a limited set of examples). These methods typically have a limited range of transmit field amplitudes in which they can accurately measure 18, 19, an effect which can be mitigated to some degree by mapping linear combinations (LCs) of channels 20, 21, 22. An alternative approach involves acquiring only a single absolute $B_{1}^{+}$ map plus a set of *relative* $B_{1}^{+}$ maps that relate the transmit field produced by each channel to the single absolute $B_{1}^{+}$ map 23, 24. The relative measurements are obtained using a low flip angle (LFA) spoiled gradient echo sequence (SPGR), for which the measured image intensity is approximately proportional to the transmit field strength. The LFA‐SPGRs are repeated, each time transmitting on a different channel or LCs, and relative transmit field maps are generated by taking appropriate image ratios.

Relative transmit field mapping relies on the low‐flip angle assumption being valid across the field of view (FOV). This assumption can prove problematic in the case of UHF transmit arrays. Very large $B_{1}^{+}$ fields can be produced directly adjacent to transmit elements, with much lower $B_{1}^{+}$ fields produced at a distance. In this situation, relative transmit field mapping becomes very challenging. Ensuring a low flip angle adjacent to a transmit element results in noise‐dominated measurements from the rest of the object where the transmit field is low in amplitude; conversely, the use of RF pulses with an amplitude sufficient to achieve adequate signal at greater distances from the coil generally results in violation of the LFA approximation near the coil.

In this work, we present a method for relative mapping of large dynamic range transmit fields. We demonstrate the utility of this method through numerical simulations, phantom imaging at 3 Tesla (T), and by use of in vivo experiments at 7T.

## THEORY

The proposed method uses a novel acquisition and reconstruction scheme. The acquisition involves the measurement of multiple SPGR images for each transmit channel, with different RF pulse amplitudes for each image so that the LFA approximation is satisfied in every voxel for at least one acquisition. The reconstruction combines the images on a voxel‐by‐voxel and channel‐by‐channel basis using prior knowledge of the SPGR signal equation to produce an image whose intensity is correctly proportional to the transmit sensitivity and is free from saturation artifact due to both the sinusoidal dependence on the flip angle and T_1_ relaxation effects. Both steps are described in the following sections.

### Acquisition

The steady‐state signal of an SPGR sequence in a voxel is given by Eq. (1) 25.

(1) $$S_{j}=\frac{M_{0}R\left(1-e^{-\frac{TR}{T_{1}}}\right)e^{-\frac{TE}{T_{2}^{*}}}e^{i\phi }\sin\left(\theta_{j}\right)}{1-e^{-\frac{TR}{T_{1}}}\cos\left(\theta_{j}\right)}+ɛ\left(\sigma \right)$$

**Figure 1:**
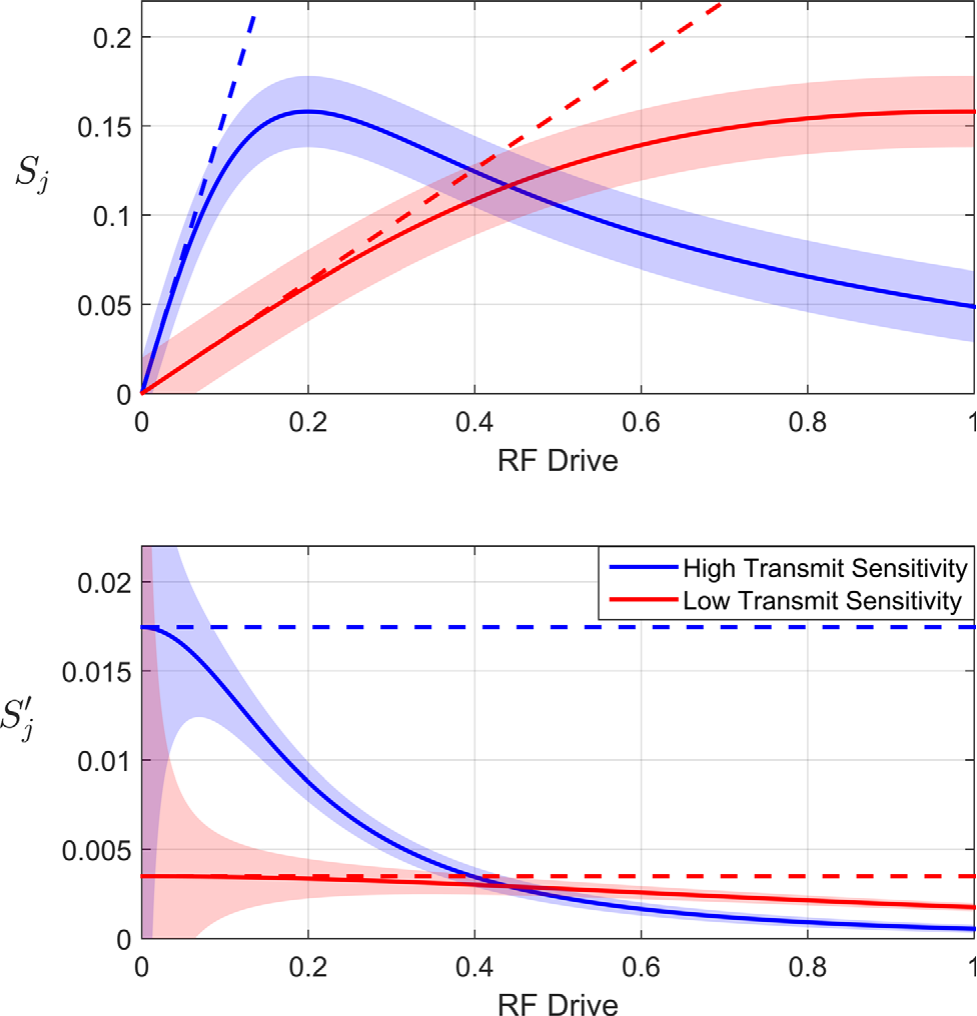
Illustrative graphs of the standard (top) and normalized (bottom) SPGR equation for the case of low (red, *f* = 0.2) and high (blue, *f* = 1) transmit sensitivities, θ_ref_ = 90°, and TR/T_1_ = 0.05. Dashed lines indicate the ideal signal in the case of no signal saturation. Shaded regions indicate ± one standard deviation.

Here, *M*_*0*_ refers to the fully relaxed magnetization at thermal equilibrium, *R* is the net sensitivity after receive channel combination, *ϕ* is the transmit phase, *TR* is the repetition time, *TE* is the echo time, and *ε* is Gaussian‐distributed noise of standard deviation *σ* (measured by a prescan) in both real and imaginary channels. The flip angle is denoted by $\theta_{j}=fd_{j}\theta_{ref}$, where *f* is the transmit sensitivity (defined as a unitless quantity giving the ratio of the actual $B_{1}^{+}$ to a reference value $B_{1,ref}^{+}$), *d*_*j*_ is the RF pulse amplitude scaling factor (also unitless, defined to lie between 0 and 1, and referred to as the RF drive), and $\theta_{ref}$ is a reference flip angle assuming a transmit field amplitude of $B_{1,ref}^{+}$ and *d*_*j*_ = 1. Finally, the index *j* denotes the *j* ^th^ repeat of *N* acquisitions with RF drive *d*_*j*_; the drives are ordered from smallest to largest. Note that *M*_*0*_, *R, T*_*1*_, $T_{2}^{*}$, *ϕ*, and *f* vary as a function of space. Figure 1A shows an illustrative plot of Eq. (1) for both high and low transmit sensitivities. Shaded regions indicate error bars at ± 1 standard deviation for an illustrative noise level *σ*.

In the LFA regime, the sine and cosine terms of Eq. (1) can be expanded using the small angle approximations sin*θ*≈*θ* and cos*θ*≈1‐*θ* ^2^/2. This results in Eq. (2), where *ρ* collects all the constant terms which are independent of the transmit channel and RF drive. The first term indicates the desired signal, which is proportional to the transmit sensitivity (dotted lines in Figure 1, top panel). The final term in Eq. (2), *η*_*j*_, accounts for systematic errors introduced by making the LFA approximation, and is present due to the dependence of *S*_*j*_ on T_1_ and flip angle; these effects are collectively referred to as saturation. It is always negative, as the SPGR curve always lies below the line of proportionality given by *ρfd*_*j*_.

(2) $$S_{j}=\rho fd_{j}+ɛ\left(\sigma \right)+n_{j}.$$

SPGR linearity is more apparent when considering the *normalized* signal, $S_{j}^{\prime }$, as defined in Eq. (3) (Figure 1, bottom panel, solid lines), obtained by dividing the raw signal by the RF drive *d*_*j*_.

(3) $$S_{j}^{\prime }=\rho f+ɛ\left(\sigma \right)/d_{j}+n_{j}/d_{j}.$$

This equation states that the normalized signal intensity is the sum of three terms. The first term *ρf* is the desired image intensity as it is proportional to the transmit sensitivity, *f*. By definition, *η*_*j*_ is negligible when in the linear regime, and so $S_{j}^{\prime }$ is independent of RF drive (Figure 1, bottom panel, dotted lines). The second term, *ε*(*σ*)/*d*_*j*_, describes contributions due to noise. The standard deviation of the normalized signal intensities are given by $\sigma_{j}^{\prime }$ = *σ*/*d*_*j*_. The last term describes the LFA approximation error, describing the discrepancy between the dashed and solid curves.

### Reconstruction

The aim of the reconstruction process is to estimate the normalized linear signal, *ρf*, given *N* measurements at drives *d*_*j*_. The proposed process to calculate the estimate is designed to use all measurements which are in the linear regime, whilst ensuring saturated measurements are rejected. This is achieved by using the statistical framework of Maximum Likelihood Estimation (MLE) 26 in conjunction with the following model:

- The first *k* measurements $S_{1}^{\prime }$, $S_{2}^{\prime }$, …, $S_{k}^{\prime }$ (1 ≤ *k* ≤ *N*) are samples drawn from probability distributions *D*(*ρf*, $\sigma_{1}^{\prime }$), *D*(*ρf*, $\sigma_{2}^{\prime }$), …, *D*(*ρf*, $\sigma_{k}^{\prime }$), all of which have the same mean, *ρf*.
- The remaining *N*‐*k* measurements $S_{k+1}^{\prime }$, $S_{k+2}^{\prime }$, …, $S_{N}^{\prime }$ are samples drawn from probability distributions *D*(*μ*_*k+1*_, $\sigma_{k+1}^{\prime }$), *D*(*μ*_*k+2*_, $\sigma_{k+2}^{\prime }$), …, *D*(*μ*_*N*_, $\sigma_{N}^{\prime }$), all of which have lower means *μ*_*k+1*_, *μ*_*k+2*_, …, *μ*_*N*_ < *ρf* due to saturation effects.

Given this model, a likelihood function $L_{k}$(*γ*| $S_{1}^{\prime }$,…, $S_{k}^{\prime }$) can be defined which gives the likelihood that a signal estimate *γ* is consistent with the first *k* measurements, as indicated in Eq. (4). The function is formed from the product of the individual sample likelihoods L(*γ*|*D*( $S_{j}^{\prime }$, $\sigma_{j}^{\prime }$)), or equivalently as the product of probability densities P( $S_{j}^{\prime }$|*D*(*γ*, $\sigma_{j}^{\prime }$)).

(4) $$L_{k}\left(\gamma |S_{1}^{\prime },\ldots ,S_{k}^{\prime }\right)=\prod_{j=1}^{k}L\left(\gamma |D\left(S_{j}^{\prime },\sigma_{j}^{\prime }\right)\right)=\prod_{j=1}^{k}P\left(S_{j}^{\prime }|D\left(\gamma ,\sigma_{j}^{\prime }\right)\right)$$

The log‐likelihood function *L*_*k*_(*γ*| $S_{1}^{\prime }$,…, $S_{k}^{\prime }$), given by Eq. (5), is used in practice as the algebra is simplified.

(5) $$L_{k}\left(\gamma |S_{1}^{\prime },\ldots ,S_{k}^{\prime }\right)=\sum_{j=1}^{k}\log\left(P\left(S_{j}^{\prime }|D\left(\gamma ,\sigma_{j}^{\prime }\right)\right)\right)$$

The proposed reconstruction scheme first finds the solution for *γ* with the maximum log‐likelihood when using *k* measurements, denoted by ${\overset{⁁}{\gamma }}_{k}$. The final image intensity, $\overset{⁁}{\gamma }$, is then obtained by selecting the ${\overset{⁁}{\gamma }}_{k}$ with maximum log‐likelihood.

Each measurement $S_{j}^{\prime }$ (1 ≤ *j* ≤ *k*) is a sample from a 2D Normal distribution N(*γ*, $\Sigma_{j}^{\prime }$), where $\Sigma_{j}^{\prime }$ = *σ'* ^2^ **I** (**I** = identity matrix). The probability density function (PDF) is given by Eq. (6).

(6) $$P\left(S_{j}^{\prime }|N\left(\gamma ,\sigma_{j}^{\prime }\right)\right)=\frac{1}{2\pi \sigma_{j}^{\prime 2}}e^{-{\left|S_{j}^{\prime }-\gamma \right|}^{2}/2\sigma_{j}^{\prime 2}}.$$

Substituting Eq. (6) into Eq. (5) produces the log‐likelihood function given by Eq. (7).

(7) $$L_{k}\left(\gamma \right)=-k\;\ln\left(2\pi \right)-\sum_{j=1}^{k}\left[2\;\ln\left(\sigma_{j}^{\prime }\right)+{\left|S_{j}^{\prime }-\gamma \right|}^{2}/2\sigma_{j}^{\prime 2}\right].$$

We now seek the value of ${\overset{⁁}{\gamma }}_{k}$ for each *k* in turn, which can be found by solving ∂*L*_*k*_(*γ*)/∂Re{*γ*} = 0 and ∂*L*_*k*_(γ)/∂Im{*γ*} = 0, resulting in:

(8) $${\overset{⁁}{\gamma }}_{k}=\frac{\sum_{j=1}^{k}d_{j}^{2}S_{j}^{\prime }}{\sum_{j=1}^{k}d_{j}^{2}}.$$

The log‐likelihoods of each ${\overset{⁁}{\gamma }}_{k}$ are evaluated to obtain the index *k*_*max*_ that yields the highest value:

(9) $$k_{max}=\underset{k}{\arg\;\max\;L_{k}\left({\overset{⁁}{\gamma }}_{k}\right)}$$

The reconstruction provides the best signal estimate $\overset{⁁}{\gamma }={\overset{⁁}{\gamma }}_{k_{max}}$ (which is a complex number that provides information on both amplitude and phase). This process is repeated serially for all voxels and transmit channels, producing images for which the signal is proportional to the transmit sensitivity. Relative transmit field maps are then obtained by taking appropriate ratios.

## METHODS

### Simulations

A Monte Carlo simulation was performed to test the effectiveness of the proposed approach. A simulated dataset was created by repeatedly modelling Eq. (1) for a wide range of different parameters.

Signals were calculated for fifty transmit sensitivities (previously denoted by the variable *f*), linearly spaced between 0.001 and 1 and a $\theta_{ref}$ = 273 °, thus ensuring that a large range of transmit conditions were explored (value of $\theta_{ref}$ chosen based on measurements from the transmit array used for imaging). TR/T_1_ ratios of 0.01 and 0.07 were simulated, with T_2_ = 100 ms and TE = 4 ms. Noise was added at SNR levels of 5%, 22%, and 100% of a reference value (SNR_ref_ = 5300). This value was obtained using pilot data taken from the in vivo acquisition described later; a ROI was drawn directly adjacent to a local receive element and divided by the standard deviation of the noise in the background. The simulated data were regenerated 50 times with different instances of noise.

Simulations were performed for 3, 4, 6, and 12 SPGR images at different RF drives. The lowest drive was selected to ensure that the maximum transmit sensitivity would produce an image signal which was linear to a 1% accuracy for each TR/T_1_ ratio. The maximum drive was defined as 1. Two different RF drive sampling schemes were used: linear, and logarithmic sampling so that more of the chosen drives are at the lower end of the range.

Each dataset was reconstructed using the proposed 2D Gaussian reconstruction. All calculations were performed in Matlab (The Mathworks Inc., Natick, MA). All of the required numerical calculations were performed using standard Matlab built‐in functions.

A complex signal estimate *η*_*a,b*_ is generated for each simulated transmit sensitivity (index a) and noise instance (index b). The average ${\bar{\eta }}_{a}$ and standard deviation $\varsigma_{a}$ of the magnitude of signal estimates across all noise instances were taken. The error metric for the signal magnitude, ${\overset{}{\bar{\delta }}}_{a}=1-\left|\frac{{\overset{}{\bar{\eta }}}_{a}}{\rho f}\right|$, and its standard deviation were examined to test the performance of the proposed method. The error metric for reconstructed phase ${\overset{}{\bar{\delta }}}_{a}^{ph}=1-\frac{∠{\overset{}{\bar{\eta }}}_{a}}{∠\rho f}$ was calculated, and its standard deviation was also assessed.

### 3T Phantom Experiments

A phantom experiment was performed to validate the proposed method against an alternative well‐established $B_{1}^{+}$ mapping technique, here chosen to be Actual Flip‐angle Imaging (AFI) with enhanced RF and gradient spoiling 14, 27. Imaging was performed on a Philips 3 Tesla (T) Achieva equipped with an eight channel PTx body coil 28. An elliptical phantom designed to mimic a torso (dimensions: 35 cm × 19 cm × 35 cm, T_1_ = 1300 ms, T_2_ = 145 ms, 0.7% salinity) was scanned in an axial orientation using a six‐channel cardiac array for signal reception. The receive channels were combined using the method proposed by Brunner 29.

AFI was performed with the following acquisition parameters: TR_1_/TR_2_/TE = 30/150/3.1 ms, field of view (FOV) = 400 × 240 mm, resolution = 5 × 5 mm, bandwidth (BW) = 1205 Hz, and number of signals averaged (NSA) = 6. The sequence was performed in slice‐selective manner (slice thickness = 10 mm), with slice‐profile effects accounted for 30. The sequence was repeated eight times to map all inverted phase linear combinations (LC) 21. The LCs were inverted to find the transmit sensitivities of each channel, $B_{1,i}^{+,AFI}$.

Further acquisitions were performed to test the proposed method. These were conducted sequentially for transmitters 2 and 7, as these channels showed the largest range of transmit sensitivities across the FOV in the previous AFI‐derived $B_{1}^{+}$ maps. Five sets of SPGR images were acquired for each transmitter; A) 16 images with linearly spaced drive levels, B) 16 images with logarithmically spaced drive levels; C) 6 images with linearly spaced drive levels; D) 6 images with logarithmically spaced drive levels; and E) 16 images acquired at the lowest drive level for subsequent averaging. Each individual SPGR image was acquired using the following sequence parameters: TR/TE = 20/3.1 ms, FOV = 400 ×240 mm, resolution = 5 × 5 mm, slice thickness = 10 mm, BW = 1205 Hz. The highest drive scale was set at the system maximum, and is here given a value of unity. The minimum drive level was selected as 0.0137, which was calculated by means of the following process. The largest transmit sensitivity of channels 2 and 7 was extracted from the previously acquired transmit field maps. Using knowledge of the TR, T1 and transmit sensitivity, the drive level was selected which produced a linear SPGR signal to an accuracy of 2.5%.

All of the data from image sets A to D were reconstructed in a channel‐by‐channel and pixel‐by‐pixel manner using the proposed MLE technique to produce the images $I_{j}^{MLE,A},\;I_{j}^{MLE,B},\;I_{j}^{MLE,C}$ and $I_{j}^{MLE,D}$. The noise level, required for the reconstruction process, was estimated from the standard deviation of signals in a manually selected background region of interest free from artifacts. Image set E was processed twice for each transmitter, first taking an average using all sixteen images (yielding $I_{j}^{ave,16}$), and second using a subset of 6 images yielding $I_{j}^{ave,6}$).

Relative transmit field maps were calculated from the AFI data by dividing the transmit field maps of channels 2 and 7 by the sum of the field maps, i.e., $rT_{j}^{AFI}=B_{1,j}^{+,AFI}/\left(\left|B_{1,2}^{+,AFI}\right|+\left|B_{1,7}^{+,AFI}\right|\right)$. These were compared with the relative transmit field maps derived from each of the four sets of MLE reconstructed images, i.e., $\;rT_{j}^{MLE}=I_{j}^{MLE}/\left(\left|I_{2}^{MLE}\right|+\left|I_{7}^{MLE}\right|\right)$ and the relative transmit field maps produced after image averaging, i.e., $rT_{j}^{ave}=I_{j}^{ave}/\left(\left|I_{2}^{ave}\right|+\left|I_{7}^{ave}\right|\right)$.

### 7T In Vivo Experiment

A proof‐of‐principle demonstration was performed in vivo. Data were acquired from a transverse slice centered on the liver of a normal volunteer on a 7T Siemens Magnetom using an eight TEM element torso transmit/receive array 31. Before the experiment, an automated coil tuning and matching procedure was performed to ensure optimal coil performance 32. B_0_ shimming was also performed.

First, a series of individual channel absolute $B_{1}^{+}$ maps were acquired to determine the maximum transmit sensitivity of each element. The prepulse FLASH technique 33, 34, 35, 36 was adapted so that both the nonselective prepulse and the slice‐selective excitation pulse were transmitted by a single element, producing a $B_{1}^{+}$ map of high SNR local to the coil. The raw images were processed to create $B_{1}^{+}$ maps, from which the maximum transmit sensitivities adjacent to the coil were extracted. The maximum of these sensitivities was used to calculate the minimum drive used for the subsequent relative $B_{1}^{+}$ mapping acquisitions. Note that this is not a necessary step to be performed on every subject; once an estimate of the maximum sensitivity for a coil array has been obtained, the appropriate minimum drive can be defined and applied for all subsequent acquisitions.

The relative mapping sequence used multiple transverse 2D SPGR sequences with the following sequence parameters: FOV = 500 × 500 mm, resolution = 3.9 × 3.9 mm, slice thickness = 8 mm, BW = 800 Hz/pixel, TR = 6ms, and TE = 2.1ms. The excitation pulse drives were scaled using the previously measured maximum local transmit sensitivity so that the maximum flip angle adjacent to any coil and hence across the FOV was 1 ° at the lowest drive level [corresponding to a SPGR small flip approximation error of <2% given the TR, assuming a T_1_ of fat at 7T (451 ms) 37]. All channels were cycled serially before acquiring at the next drive level, as pilot data demonstrated that this was the most effective means to circumvent spin history effects from neighboring coils. Four drive levels were acquired using logarithmic drive sampling. The maximum drive level was again chosen as the system maximum. The total duration of the calibration sequence (32 images in total) was 22 s and was achieved within a breath‐hold by not using dummy scans or placing gaps between acquisitions.

The measured data were exported and Fourier transformed. Receive channels were combined using an SVD‐based approach 29. These were then reconstructed using both the proposed method described in the Theory section. Relative transmit field maps were produced by dividing each reconstructed image by the sum of the magnitudes of all reconstructed images. The reconstruction times on a standard desktop PC times were under a second per transmit channel.

Reconstruction code can be found online at http://mriphysics.github.io.

## RESULTS

### Simulation

**Figure 2:**
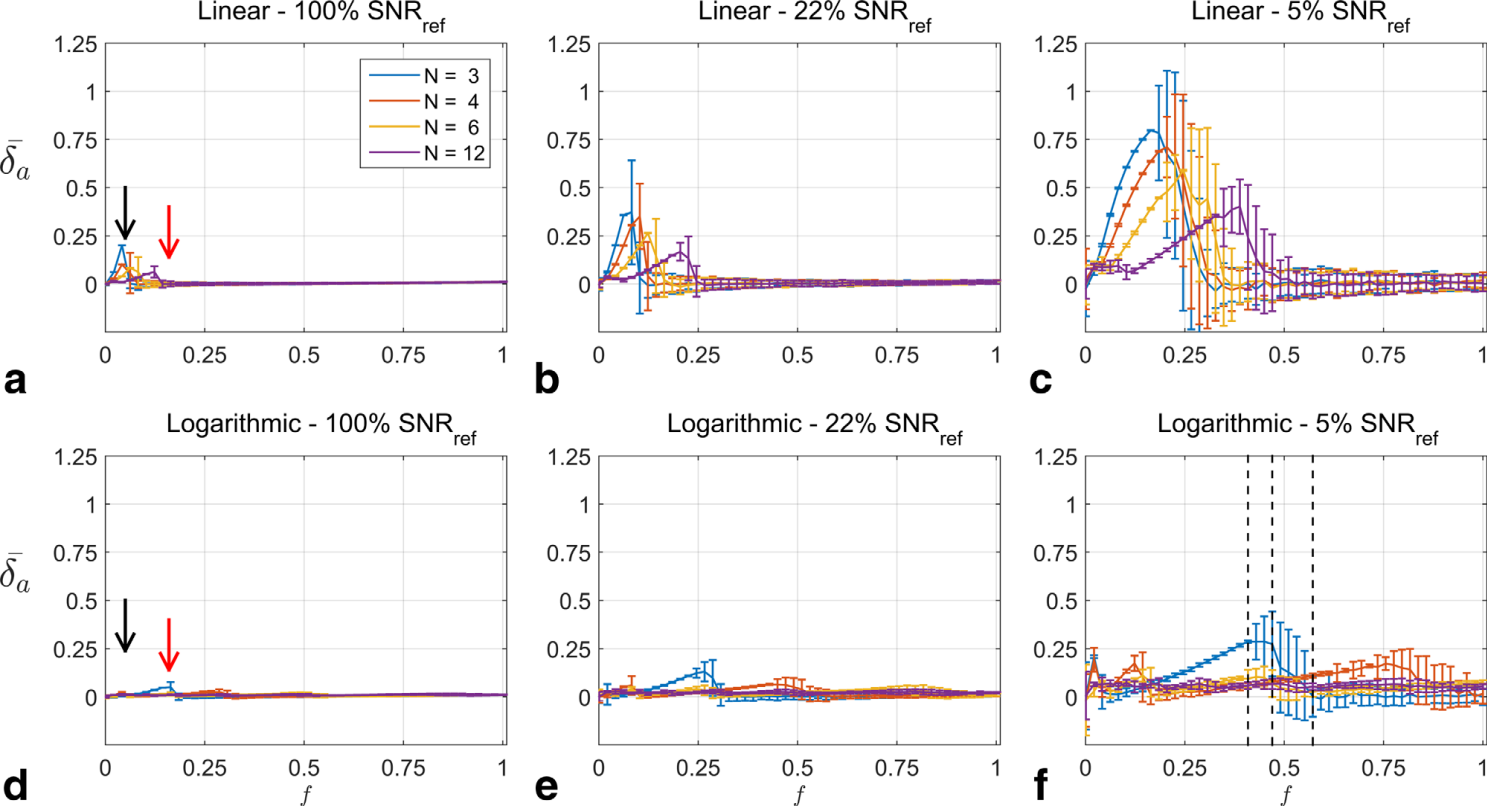
**a–f**: Results of numerical simulations comparing linear drive sampling (top row) and logarithmic drive sampling (bottom row) at 100% (left column), 22% (center column), and 5% (right column) of SNR_ref_. Each subplot has the transmit sensitivity on the horizontal axis and error on the vertical axis. Different colored lines indicate different number of measurements. The black and red arrows indicate aspects of the figure discussed further in the text.

Figure 2 shows graphs comparing linear drive sampling (top row) with logarithmic drive sampling (bottom row) at all SNR levels and for all transmit sensitivities. The displayed results are for the case TR/T1 = 0.01; the results for TR/T1 = 0.07 are omitted as the same behavior is seen as described below. Results displayed are restricted to 2D Gaussian signal amplitude reconstruction. Each graph shows the error ${\overset{}{\bar{\delta }}}_{a}$; perfect reconstructions correspond to ${\overset{}{\bar{\delta }}}_{a}=0$. Error bars correspond to the standard deviation of ${\overset{}{\bar{\delta }}}_{a}$ across all noise repeats. Differently colored lines indicate different numbers of measured drive scales (N), as indicated by the legend.

In the case of 100% SNR_ref_, both sampling schemes perform well over a wide range of transmit sensitivities (0.2 < f < 1, ${\overset{}{\bar{\delta }}}_{a}$ < 3%). In the region f < 0.25, linear sampling has larger maximum errors than logarithmic sampling for all N (black arrows, Figures 2A,D). Logarithmic sampling can perform more poorly than linear sampling at certain transmit sensitivities (i.e., N = 3, red arrows in Figures 2A,D), but the size of the error is smaller than that of linear sampling at lower transmit sensitivities. Furthermore, increasing N yields greater reconstruction quality improvements for logarithmic sampling over linear sampling (i.e., error curves for N = 12 are lower for logarithmic over linear sampling).

At 22% SNR_ref_ (Figs. 2B,E), logarithmic sampling again outperformed linear sampling for low transmit sensitivities (f < 0.25). This comes at the expense of the reconstruction quality at higher transmit sensitivities (0.25 < f < 0.6), for which there is a small degree (<10%) of signal underestimation when N = 3 or N = 4. However, as seen earlier at 100% SNR_ref_, increasing N allows for these errors to be eliminated.

These effects are even more pronounced at the lowest SNR level (Figs. 2C,F). Linear sampling produces highly erroneous reconstructions for *f* < 0.5, yet accurate reconstructions above it. Logarithmic sampling produces high quality reconstructions if N ≥ 6; reducing N results in erroneous reconstructions but which are still superior to those produced by linear sampling.

**Figure 3:**
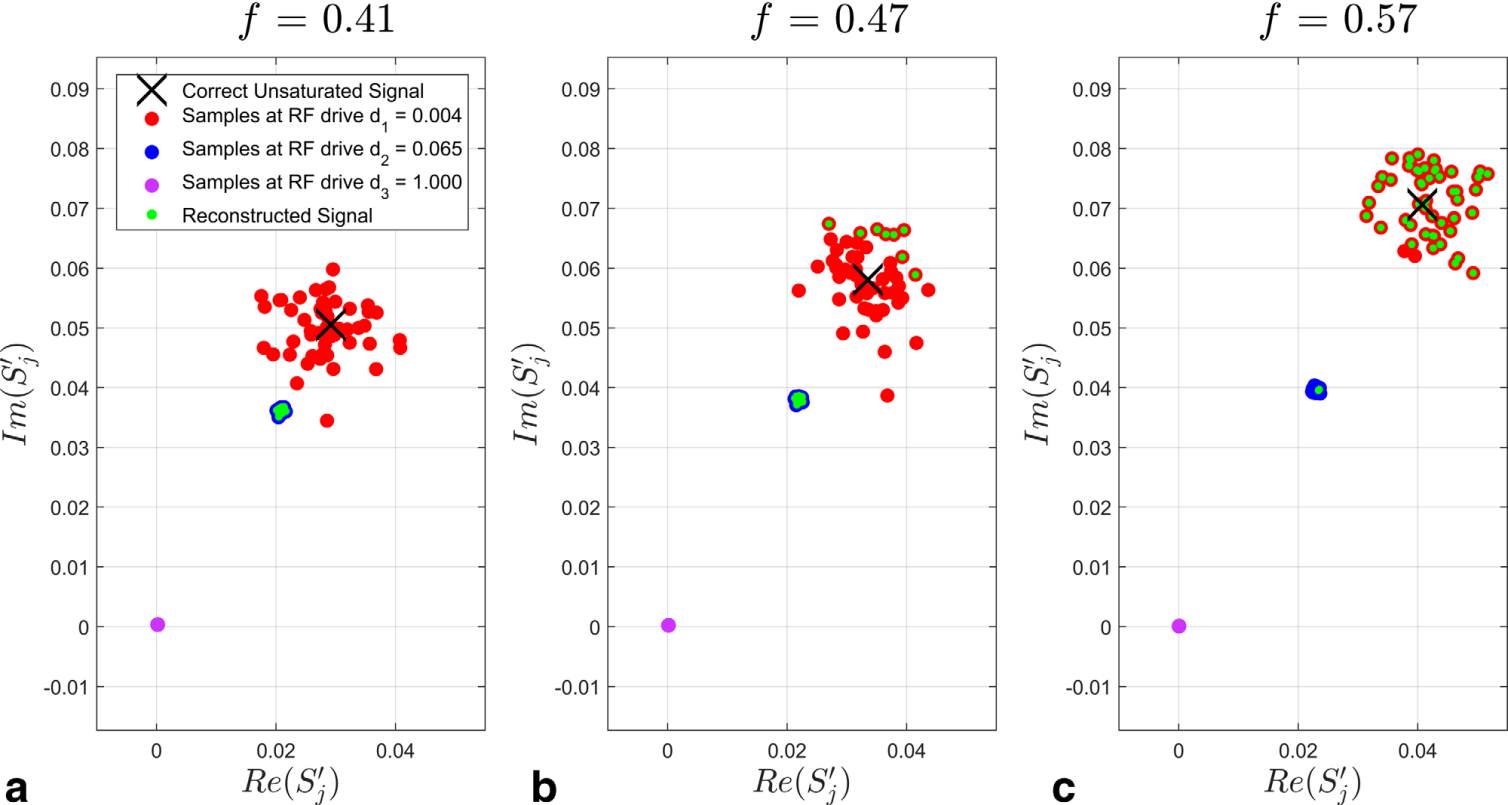
**a–c**: Demonstration of reconstruction behavior at three different transmit sensitivities in the case of logarithmic sampling. Each axis shows the complex plane of SPGR signals after normalization. Black cross: correct unsaturated signal; red dots: samples at first RF drive level (d_2_ = 0.004); blue dots: samples at second RF drive level (d_1_ = 0.065); purple dots: samples at third RF drive level (d_3_ = 1.000); green dots: reconstructed signal estimates. The signals at the third drive level are at the origin due to signal saturation.

A prominent feature in the Figure 2 is the change in standard deviation with transmit sensitivity. For example, consider the N = 3 curve in Figure 2F. The error bars transition from narrow to wide at *f* = 0.42, which is also the location of maximum error. This behavior is elucidated in Figure 3, which shows the reconstruction results for each noise repeat at the three transmit sensitivities indicated by vertical dashed black lines in Figure 2F.

At *f* = 0.41 (Figure 2F, left vertical dashed line), the result is inaccurate (as ${\overset{}{\bar{\delta }}}_{a}$ is non‐zero) yet precise (narrow error bars). The reason for this is shown in Figure 3A. The correct signal is shown by the black cross; the noisy samples at the three drive levels are given by the red, blue, and purple dots, and the reconstructed signal given by the smaller green dots. At this transmit sensitivity, the reconstruction cannot determine that the samples at drive level 2 are saturated as the standard deviation at the first RF drive level is too large. Therefore the reconstruction selects the measurements at the second drive level; these are incorrect, but the standard deviation of the result is small as the spread of the data is inversely proportional to the RF drive level.

Figure 3B explains the behavior at *f* = 0.47 in Figure 2F where the result is both inaccurate and imprecise. The reconstruction behaves in either one of two ways: first, the reconstruction can produce an estimate very close to the second sample (as in Figure 3A) or, secondly, when the first sample is sufficiently different from the second simply due to noise, the reconstruction only uses the first sample. Therefore the average across all noise instances is incorrect, and the resulting standard deviation is very large.

The behavior of the reconstruction beyond this transition region is shown in Figure 3C. Here, the transmit sensitivity is sufficiently large that the reconstruction can exclude saturated measurements for almost all noise instances. The average across all noise instances is therefore accurate, and the imprecision reflects the SNR of the system.

**Figure 4:**
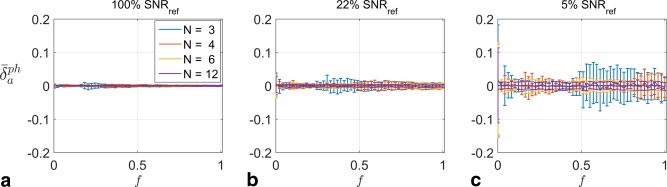
**a–c**: Signal phase estimation error for the three SNR scenarios using logarithmic drive sampling.

Figure 4 shows the error when estimating the phase of the signal. There is little bias in the estimation (as all curves lie on ${\overset{}{\bar{\delta }}}_{a}^{ph}$ = 0), and the variation of error bars with transmit sensitivity can be explained by the same mechanism shown in Figure 3.

### Phantom Results

**Figure 5:**
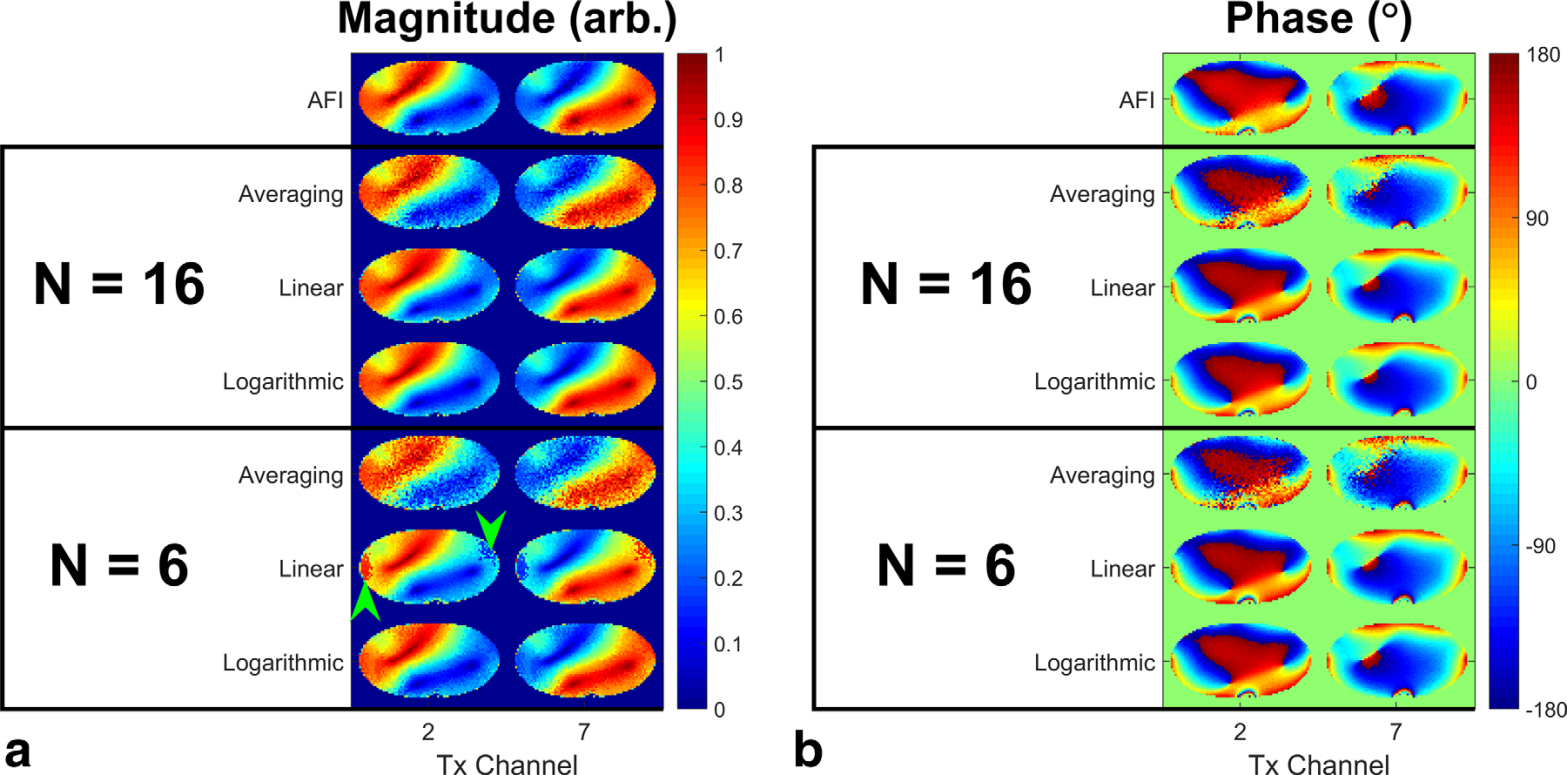
Relative $B_{1}^{+}$ map magnitudes (**a**) and phases (**b**) acquired on a 3T PTx system. Top row: results using AFI; subsequent rows: relative maps estimated using different quantities of SPGR data, reconstruction schemes and RF drive level sampling patterns. Green arrowheads indicate features discussed in the main body of the study.

The phantom results are shown in Figure 5. Figure 5A shows the relative $B_{1}^{+}$ maps from all experiments. Relative transmit field maps obtained by averaging 16 SPGR images at the lowest drive level are severely affected by noise. Using either linear or logarithmic drive sampling and 16 measurements yield relative transmit field maps which are consistent with those produced by AFI. Reducing the number of measurements to six results in very noisy relative transmit field maps if using solely averaging. Maps derived using linear sampling suffer from artifacts (green arrowheads) where there is insufficient information to accurately estimate the unsaturated signal. However, using logarithmic sampling still produces maps which are consistent with the AFI measurements. Figure 5B displays the reconstructed phases of all phantom experiments. Reconstructions using the proposed MLE method produce phase maps which are consistent with the AFI maps. Phase maps produced by averaging SPGR images are visibly affected by noise.

### In Vivo Results

**Figure 6:**
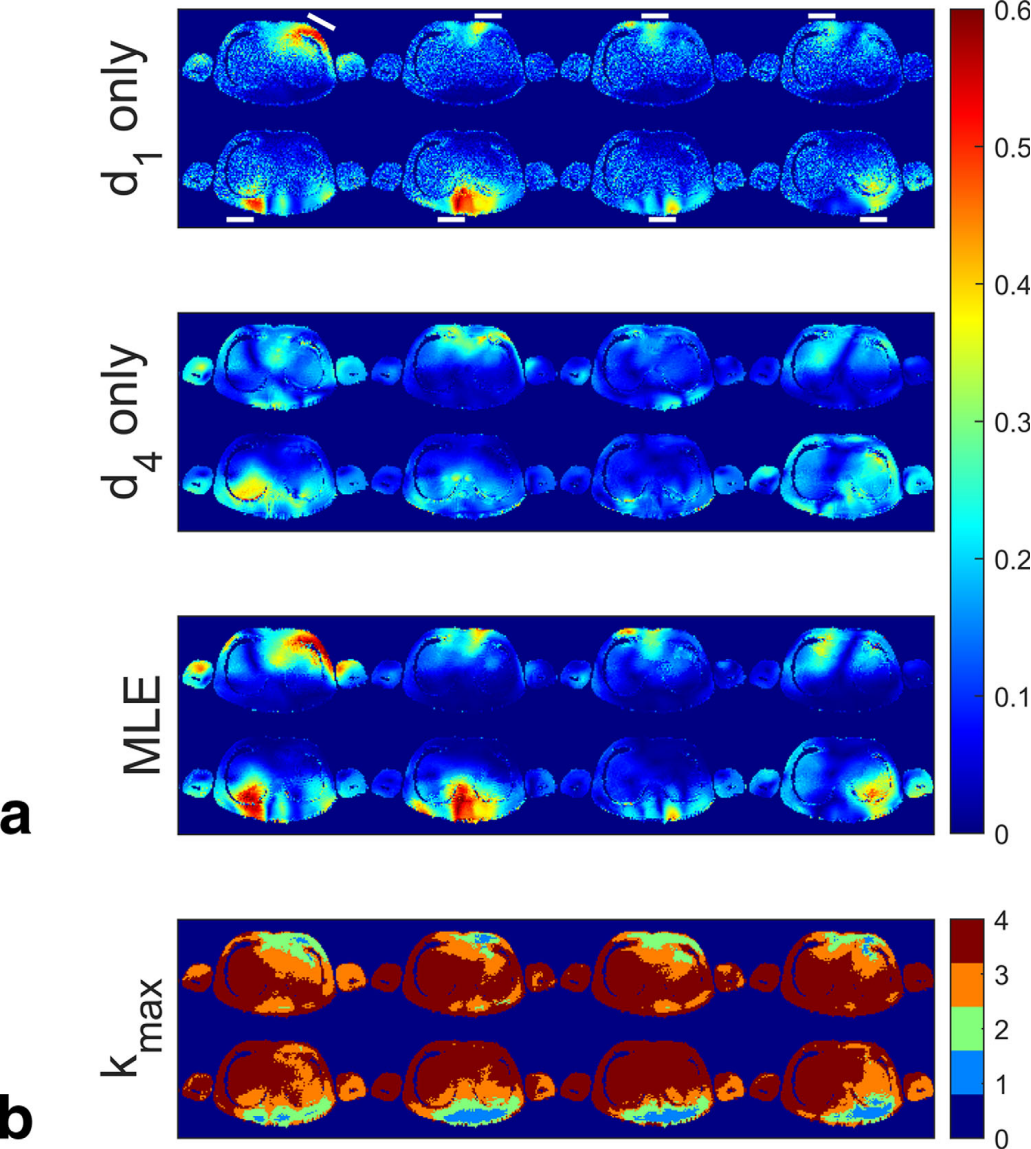
In vivo relative $B_{1}^{+}$ maps. **a**: Relative map magnitude when using data acquired using the first drive level (top panel), last drive level (center panel), and generated by the MLE reconstruction (bottom panel). Coil positions are indicated by the white lines in the top panel. **b**: Number of measurements used for the MLE reconstructions. k_max_ can take integer values between 1 and 4.

Figure 6A shows the amplitude of the relative $B_{1}^{+}$ maps calculated from the in vivo data. The top panel shows the maps calculated using images from the first RF drive level only. The SNR at the center of the maps is poor as the RF drive must be low to maintain the validity of the LFA approximation adjacent to the coils. The middle panel shows the relative maps calculated from the images obtained with the largest drive. There is sufficient SNR to calculate the maps, but the signal amplitude estimates at the edges of the subject are incorrect because the LFA is not valid in these regions at this drive level. The relative $B_{1}^{+}$ maps produced using the proposed method provide good estimates across the FOV (bottom panel). They agree with the relative maps calculated from the first drive level at the edge of the subject close to the transmit elements, and also agree with the highest voltage maps in the regions where the signal is linear for all images and all channels.

Figure 6B shows the number of measurements used to recover the best signal estimate for each pixel. Lower numbers of samples are used in the close vicinity of transmit elements (e.g., channel 7, transmit element is below the subject), and larger numbers of samples are used remote to the transmit elements.

**Figure 7:**
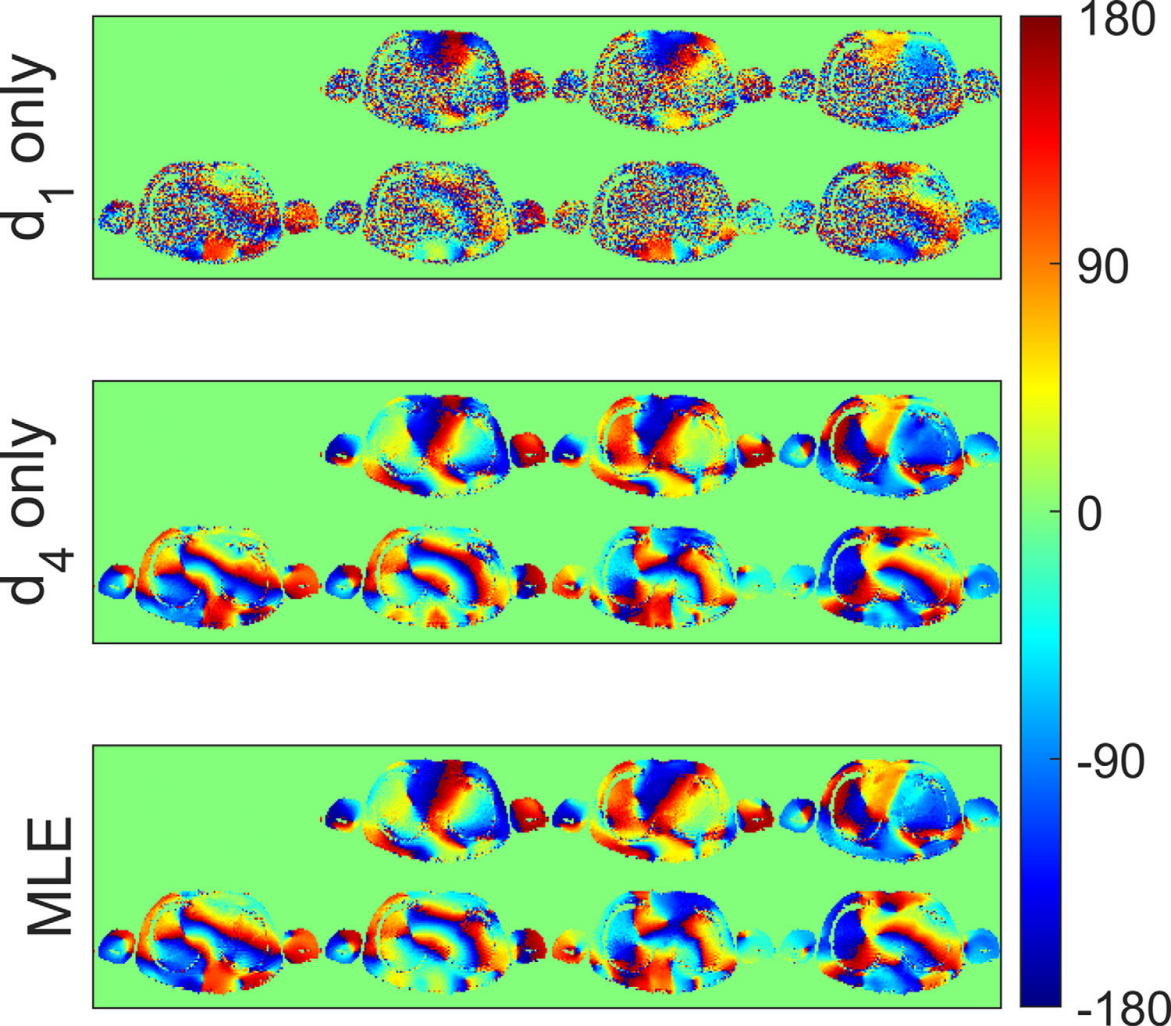
Relative $B_{1}^{+}$ phase maps. Top panel: drive level 1 only; middle panel; drive level 4 only; bottom panel; phase estimated by MLE reconstruction.

Figure 7 shows the relative channel phases of the transmit elements. As before, the lowest drive levels produce noisy maps remote from the transmit elements. The MLE reconstruction produces high SNR phase estimates across the FOV.

## DISCUSSION

We have demonstrated an acquisition scheme and reconstruction method which can produce images whose intensity and phase is proportional to applied transmit field in the presence of large dynamic range. This enables the accurate measurement of relative transmit field maps when there are both high and low transmit field amplitudes present in the imaged FOV. The method was tested using Monte Carlo simulations at multiple SNR levels, verified at 3T by comparing it to an alternative $B_{1}^{+}$ mapping method, and demonstrated in vivo at 7T.

Both the numerical simulations and phantom results demonstrated that using more images produces superior reconstructions. However, this will come at the expense of measurement time. The results also showed that logarithmic sampling produced superior results for the majority of transmit sensitivities, and therefore this was applied in vivo. However, alternative voltage sampling schemes are possible, and further optimization will be the subject of future work.

Care must be taken when designing $B_{1}^{+}$ mapping methods to make the measurement insensitive to relaxation effects. The main impact of T_1_ on the method proposed here is determining the shape of the SPGR signal curve, and hence determining at which point the signal leaves the linear regime. The relative $B_{1}^{+}$ maps produced in vivo show very little tissue‐dependent contrast, giving confidence that the method is robust to T_1_ variations. This is also supported by further numerical simulations (not presented in the study) at different TR/T_1_ ratios, which also showed that the method is insensitive to relaxation effects.

It is also possible to alter the formulations of the likelihood functions. One further extension would be to use neighborhood pixels to increase the statistical power of the MLE, or to estimate the correct signals for all transmitters simultaneously to exploit the spatial smoothness of the transmit field 38, 39.

Although 2D imaging was used here, the approach equally applies to multislice and 3D volumetric mapping. Furthermore, the specific MR sequence used here was designed for the TEM torso array used in this work. Here we could perform reliable mapping acquiring data with four RF drive levels; other arrays with different geometries may require more or less measurements. There will also be a field‐strength dependence to the number of required measurements; lower field strengths will require fewer measurements, larger field strengths more. Furthermore, this work used an absolute mapping precalibration stage to determine the maximum $B_{1}^{+}$ produced by each coil. This does not need to be performed on every subject, as the used array can be tested on many different loads to measure the maximum transmit field it can produce. Subsequent subject scanning can then use a minimum drive scale designed with this maximum sensitivity. The highest drive level was chosen as the maximum deliverable by the used MR system, as this allows the best mapping of the $B_{1}^{+}$ voids generated by the transmit array. However, this may not be the best choice for arrays which have large dynamic range yet no $B_{1}^{+}$ voids, as this could result in saturation across the entire FOV.

The notion of using multiple different RF pulse voltages to extend $B_{1}^{+}$ mapping dynamic range has only been proposed previously in the context of absolute $B_{1}^{+}$ mapping 38, 40. The method proposed here is devoted to relative transmit field mapping.

This study does not directly address the question of how to produce absolute $B_{1}^{+}$ maps in the presence of fields with large dynamic range. All absolute $B_{1}^{+}$ mapping methods have a limited range of transmit sensitivities in which they can obtain reliable measurements 18, 19, 41. At high field, it is often difficult to find a single $B_{1}^{+}$ shim for which the dynamic range is sufficiently small, even when using linear combinations 20, 22, 35. Several recently proposed approaches enable absolute $B_{1}^{+}$ map estimation without direct measurement by making simplifying assumptions about the data; for example, assuming that the sum of the magnitude of the transmit fields equal the sum of the magnitudes of the receive fields 42, or assuming that the RF fields can be expressed as a sum of Bessel functions 43. These two approaches rely on having SPGR images for which the signal is proportional to the transmit field. The method proposed in this study provides SPGR images with this property; therefore, unifying these methods could be the subject of future work.

In conclusion, this study presents a new method for relative transmit field mapping in the presence of large transmit dynamic range. In the presented in vivo demonstration, full relative mapping of eight transmit channels was achieved for a single slice in a breath‐hold.
